# Age-Dependent Remodeling in Infrapatellar Fat Pad Adipocytes and Extracellular Matrix: A Comparative Study

**DOI:** 10.3389/fmed.2021.661403

**Published:** 2021-05-10

**Authors:** Elena Stocco, Elisa Belluzzi, Martina Contran, Rafael Boscolo-Berto, Edgardo Picardi, Diego Guidolin, Chiara Giulia Fontanella, Eleonora Olivotto, Giuseppe Filardo, Giulia Borile, Filippo Romanato, Roberta Ramonda, Pietro Ruggieri, Marta Favero, Andrea Porzionato, Raffaele De Caro, Veronica Macchi

**Affiliations:** ^1^Department of Neuroscience, Institute of Human Anatomy, University of Padova, Padova, Italy; ^2^L.i.f.e.L.a.b. Program, Consorzio per la Ricerca Sanitaria, Padova, Italy; ^3^Orthopedic and Traumatologic Clinic, Department of Surgery, Oncology and Gastroenterology, University of Padova, Padova, Italy; ^4^Musculoskeletal Pathology and Oncology Laboratory, Department of Surgery, Oncology and Gastroenterology, University of Padova, Padova, Italy; ^5^Centre for Mechanics of Biological Materials, University of Padova, Padova, Italy; ^6^Department of Industrial Engineering, University of Padova, Padova, Italy; ^7^RAMSES Laboratory, Research, Innovation & Technology (RIT) Department, Istituto di Ricovero e Cura a Carattere Scientifico (IRCCS) Istituto Ortopedico Rizzoli, Bologna, Italy; ^8^Applied and Translational Research Center, Istituto di Ricovero e Cura a Carattere Scientifico (IRCCS) Istituto Ortopedico Rizzoli, Bologna, Italy; ^9^Department of Physics and Astronomy “G. Galilei,” University of Padova, Padova, Italy; ^10^Institute of Pediatric Research Città della Speranza, Padova, Italy; ^11^Rheumatology Unit, Department of Medicine, University - Hospital of Padova, Padova, Italy; ^12^Internal Medicine 1, Ca' Foncello Hospital, ULSS2 Marca Trevigiana, Treviso, Italy

**Keywords:** infrapatellar fat pad, aging, osteoarthritis, anterior-cruciate ligament rupture, cadaver donors, extracellular matrix

## Abstract

The infrapatellar fat pad (IFP) is actively involved in knee osteoarthritis (OA). However, a proper description of which developmental modifications occur in the IFP along with age and in absence of joint pathological conditions, is required to adequately describe its actual contribution in OA pathophysiology. Here, two IFP sources were compared: (a) IFP from healthy young patients undergoing anterior-cruciate ligament (ACL) reconstruction for ACL rupture (*n* = 24); (b) IFP from elderly cadaver donors (*n* = 23). After histopathological score assignment to confirm the absence of inflammatory features (i.e., inflammatory infiltrate and increased vascularity), the adipocytes morphology was determined; moreover, extracellular matrix proteins were studied through histology and Second Harmonic Generation approach, to determine collagens content and orientation by Fast Fourier Transform and OrientationJ. The two groups were matched for body mass index. No inflammatory signs were observed, while higher area, perimeter, and equivalent diameter and volume were detected for the adipocytes in the elderly group. Collagen III displayed higher values in the young group and a lower total collagen deposition with aging was identified. However, collagen I/III ratio and the global architecture of the samples were not affected. A higher content in elastic fibers was observed around the adipocytes for the ACL-IFPs and in the septa cadaver donor-IFPs, respectively. Age affects the characteristics of the IFP tissue also in absence of a pathological condition. Variable mechanical stimulation, depending on age-related different mobility, could be speculated to exert a role in tissue remodeling.

## Introduction

Age represents the strongest predictor of development for the most common musculoskeletal disorder worldwide: osteoarthritis (OA) (1–4), a disease of great impact, especially in the light of the World Population Prospects report data (5) dreading a great shift in demographics. To date there are about 962 million individuals over the age of 60 which will rise, reaching 1.4 billion in 2030 and 2.1 billion in 2050. When referring to individuals of 80 years old or over (today 137 million), a growing of 32.2% is expected for 2050 (5).

To figure out the role of a tissue in a pathological condition, a fine understanding of its physiological-developmental characteristics is mandatory. Homeostasis maintenance is an age-dependent event; according to a biological perspective, from aging descends a progressive decline in tissue physiological homeostasis thus provoking an increased impaired function. For example, age-related mobility disability has largely been attributed to compromised skeletal muscle characteristics (6, 7) or cartilage alterations (2). However, also adipose tissue features (e.g., cellular composition and secreted factors) likely play a critical role in this process (6) and, considering synovial joints, adipose tissue is both recognized in the sub-synoviocyte intimal layer and in the periarticular depots of the knee joint, like the infrapatellar fat pad (IFP) that specifically consists in white adipose tissue (8).

The IFP has recently gained attention for its active contribution in OA (9, 10). Thus, beyond the classical description recognizing it as a mere shock absorber, today the IFP is identified as a local source of adipocytokines (11) in continuity with the synovial membrane and actively involved in a cross-talk with the surrounding environment (12–15).

Awareness about IFP age-dependent remodeling, in the absence of signs of pathology, may help in a fine description of the mechanisms underlying OA, with insights about its contribution to the disease. Many authors previously featured subcutaneous adipose tissue as a control for IFP; however, a histo-topographic study by Macchi et al. (8), followed by a biomechanical analysis (16), challenged this assumption, highlighting the existence of differences between the IFP and other sources of adipose tissue (i.e., knee and abdomen), due to both anatomical origin and function. In fact, the vulnerabilities of a joint that occur as part of the aging process make it susceptible to disease (17).

Aging and disease are not necessarily synonymous; however, age-associated alterations in tissue structure and function could partly represent the link that explains the risky component of aging (18). Here, for the first time to our knowledge, we aim to verify which modifications/alterations occur in the IFP along with aging, in case of the absence of cartilage defect. Therefore, it can be hypothesized that: (a) in case of no cartilage lesions, the IFP will not show inflammatory features; (b) a variation in adipocytes area is expected according to the age of the patients/donors, despite maintenance of the typical cell morphology; (c) a reduction in characterizing matrix proteins as well as a different distribution are plausible with aging.

Providing a broad description of IFP morphological variations correlated with aging will help to identify the key elements for a rigorous understanding of IFP role in OA. To address this purpose, the IFPs isolated from patients undergoing anterior cruciate ligament (ACL) reconstruction for ACL rupture (young group) were compared with those of cadaver donors from “Body Donation Programs” (elderly group), to assess specific features at both cellular level and extracellular matrix (ECM) compartment.

## Materials and Methods

### Study Groups Characteristics

Two different groups were considered in this study: the young group and the elderly group. For the young group, 24 healthy patients with chronic ACL rupture undergoing arthroscopic ACL reconstruction were enrolled at the IRCSS Rizzoli Orthopedic Institute (Bologna, Italy) after providing written informed consent; small biopsies of IFP were obtained during arthroscopic surgery. In accordance to the inclusion/exclusion criteria, patients were without tumor and/or knee comorbidities, previous knee surgery or other pathologies, and not suffering from infective conditions, coagulation disorders, rheumatic, inflammatory, or metabolic diseases. All patients had no cartilage defects (*n* = 20 patients, Outerbrigde Grade 0; *n* = 4 patients, Outerbridge Grade 1). Visual analog scale (VAS) was recorded to evaluate pain.

The IFP samples belonging to the elderly group were collected from 23 not-embalmed cadavers enrolled in the Body Donation Program “Donation to Science” of the University of Padova (19–21). Donors did not have any history of symptomatic OA, or knee comorbidities as well as rheumatic or inflammatory disorders/tumors or signs of cartilage degradation or presence of osteophytes on dissection. Full longitudinal thickness samples of IFP were sampled according to a protocol previously described (8). The IFP samples/biopsies were processed for histological/immunohistochemical analysis and ultrastructural characterization studies as described below.

### IFP Histopathological Score Assignment

Immediately after excision, IFP biopsies/samples were fixed in 10% formalin solution and paraffin embedded according to routine protocols. Thereafter, to verify the possible occurrence of an inflamed status, IFP score based on the presence of lymphocytic infiltration and vascularity was determined, as previously described (10). Briefly, 10 μm thick sections (three consecutive sections) were stained with hematoxylin-eosin (H&E). The presence of mononuclear cell infiltration was evaluated and graded as follows: grade 0 = no presence of lymphocytic infiltration; grade 1 = presence of perivascular mononuclear cell infiltration; grade 2 = both perivascular and interstitial mononuclear cell infiltration. Vascularity was also evaluated on H&E sections, counting the total number of vessels in three sequential sections for each case. The images were recorded with a high-resolution digital camera (DC 200, Leica Microsystems).

### Adipocytes Morphometric Study

Morphometric analysis was developed on full-thickness specimens, as previously described (8, 22). Briefly, 10 μm thick sections (three consecutive slices/sample), stained with H&E, were recorded with a high-resolution digital camera (DC 200, Leica Microsystems). With a data processing software (Matlab R2019b, MathWorks Inc., Natick, MS, USA), the adipocytes were selected, and the images were converted to 8-bit binary images. The perimeter (i.e., the distance around the boundary of the selected region) and the area of the internal selected region were determined for each adipocyte. Hence, the equivalent diameter (i.e., the diameter of a circle with the same area as the region) was determined, and the equivalent volume was calculated. Considering the adipocytes morphometry, the adipocytes were then automatically approximated to ellipses prior to calculate major and minor axes and eccentricity (23, 24). For each section, three different fields were analyzed.

### Extracellular Matrix Characterization

#### Histological Evaluation: Collagen and Elastic Fibers Content

Sirius red and Weigert-van Gieson stainings were adopted to characterize the IFP ECM. In particular, Sirius red staining allowed to identify the presence of collagen subtypes: type I and type III collagens were distinguished under polarized light microscope (Leica DMR microscope, Leica Microsystems, Wetzlar, Germany) appearing orange to red and yellow to green, respectively (8). Van Gieson staining specifically identified the elastic fibers content. Thus, the characterization of the pericellular areas and septa with reference to the types of collagen and elastic fibers were both considered in the two groups.

Quantification of collagen (type I and III) and elastic fibers occurred according to a protocol previously developed (8), using ImageJ software (at http://rsb.info.nih.gov/ij/) (25). Briefly, for type I and III collagen content, the sections were analyzed considering three fields for each group. Images were acquired in polarization light at 5X magnification and saved as TIFF files, thereafter red-orange and green-yellow areas were identified by setting adequate thresholds of the “hue” component. Collagen composition was determined according to red-orange and green-yellow ratio. Also, for the elastic fibers three random fields per section were selected and full-color images were acquired at a 20X magnification. The analyses were then performed on TIFF files after applying color thresholding to identify the elastic fibers. Stained structures were identified by selecting the pixels with a “hue” in the blue-violet range and “brightness” lower than the mean brightness level exhibited by the background minus 2 standard deviations (SD) (26). An arbitrary unit (AU) was used to show the amount of the considered proteins (i.e., collagens and elastin).

#### Multiphoton Microscopy: Total Collagen Content and Fibers Orientation

Second Harmonic Generation (SHG) imaging was performed through a custom developed multiphoton microscope, previously described by Filippi et al. (27). Briefly, an incident wavelength of 800 nm (~40 mW average laser power, under the microscope objective) was adopted to detect the collagen's SHG signal at 400 nm.

The images were acquired at a fixed magnification through the Olympus 25X water immersion objective with 1.05 numerical aperture (1,024 × 1,024 pixels), averaged over 100 consecutive frames, with a pixel dwell time of 0.14 μs and a pixel width of 0.8 μm.

To describe organization and distribution of the fibers within the sample, coherency was determined through Fast Fourier Transform (FFT) and OrientationJ. The FFT of an image is used to estimate its spectrum and detect preferential fibers direction. If the image is highly oriented in a single direction, it results in an elliptic shape; if the components of the image are spread in all directions, the FFT results in a circular shape (28). The power plots of FFT in different samples was fitted with an ellipse and the ratio between long and short axis was calculated for each plot. OrientationJ is a Java plugin for ImageJ/FIJI which allows to visualize with a Hue, Saturation and Brightness (HSB) map the directionality within the sample. Every color is assigned to an orientation angle and the presence of all colors in the images means that no preferential orientation is present (29). Five samples for the young-IFP group and four for the elderly-IFP group were considered in this analysis; for each sample, 5 different areas were analyzed.

### Statistical Analysis

Results were reported as the mean and range or median and interquartile range (IQR). The Shapiro-Wilk test was used to determine whether data were distributed normally. Chi-square (χ^2^) test or Fisher's exact test were performed to compare categorical and dichotomous data. Mann-Whitney test or unpaired *t*-test were used to compare continuous variables. Correlations were performed by Spearman's coefficient to identify association between variables. Data were analyzed using SPSS version 25.0 for Windows (IBM, Armonk, NY, USA) or GraphPad 6 (San Diego, USA). The significance level (*p*) was set at 0.05 (*p*-value < 0.05).

## Results

### Demographic Data of the Two Study Groups

The median age of the ACL IFP group was 32.5 years (IQR, 42–22) (females, *n* = 6; males, *n* = 18), while the median age of IFP cadavers was 74 years (IQR, 83–66) (females, *n* = 12; males, *n* = 11) (*p* < 0.0001). All patients were Caucasians. The time between injury and surgery of patients with ACL rupture was at least 6 months (median 8 months; IQR, 14.5–6). The median body mass index (BMI) Kg/m^2^ was 22.81 (IQR, 24.58–20.50) for ACL patients and 23.03 (IQR, 29–22.42) for cadavers (*p* = 0.106). ACL IFP group reported a median VAS value of 1 (IQR, 5–0).

### IFP Histopathological Grading: Absence of OA Signs

**Figure 1:**
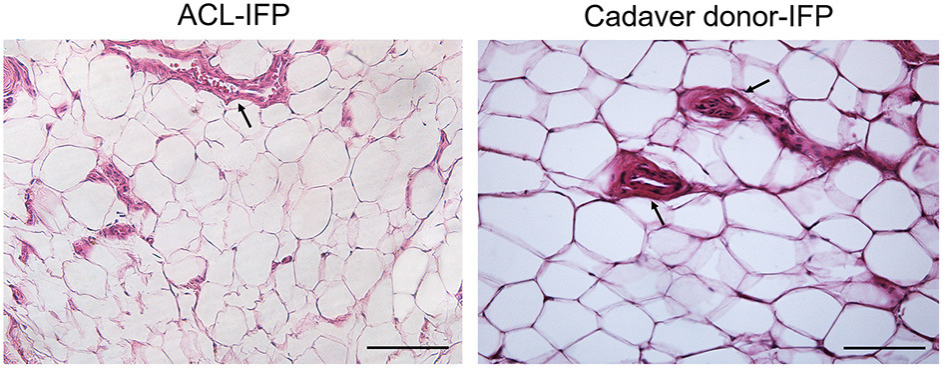
Inflammatory infiltration and vascularity. No lymphocytic infiltration was detected in both groups. H&E staining showing vessels distribution (black arrows) in ACL-IFP and cadaver donors-IFP (scale bars=100 μm). Vascularity is comparable between the two experimental groups. In both groups it is clearly recognizable the cross-section of the blood vessels lumen, lined by the endothelial cells.

H&E staining allowed to perform the histopathological grading of the IFP tissues (Figure 1). For both lymphocytic infiltration and vascularity, no significant differences were observed between the two groups. All IFPs showed grade 0 of lymphocytic infiltration except for one ACL-IFP, which was graded as 1 (3.6%) (*p* = 0.312). The median values recorded for vessels were 8.9 (IQR, 17.5–7.2) for ACL-IFPs and 12.1 (IQR, 14.42–8.94) for cadaver donors (*p* = 0.651) (Table 1).

**Table 1:** IFP histopathological grading.

	**ACL^a^ patients**	**Cadavers**	***p*-value**
	**(*n* = 23)**	**(*n* = 23)**	
**Lymphocytic infiltration, number (%)**			
GRADE 0	22 (95.7%)	23 (100%)	0.312
GRADE 1	1 (4.3%)	0	
GRADE 2	0	0	
Vascularity, median (IQR)^b^	8.9 (17.5–7.2)	12.1 (14.42–8.94)	0.651

a *ACL, anterior cruciate ligament;*

b *IQR, interquartile range*.

### Morphometric Study Showing Smaller Adipocytes Area in Young vs. Elderly Group

**Figure 2:**
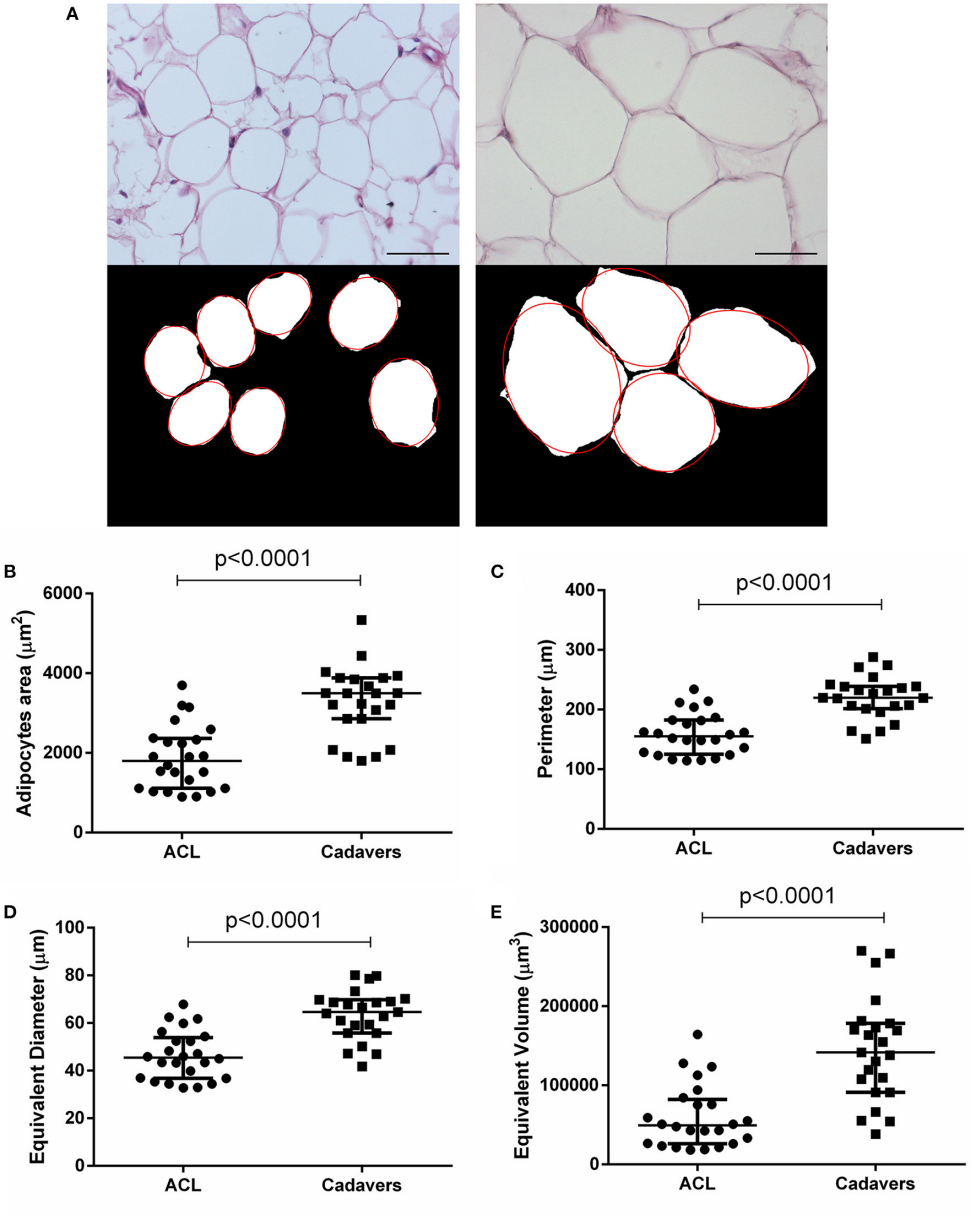
Adipocytes morphometric analysis. **(A)** After H&E staining of ACL-IFPs (scale bar = 100 μm) and cadaver donor-IFPs (scale bar = 200 μm), the images were processed. The adipocytes were manually identified (see the red-line profile); thus, the images were converted into the corresponding 8-bit gray-level images for the measurements of mean adipocytes area (μm^2^). As showed in the graphs, a significant difference (*p* < 0.0001) was detected between the two experimental groups for the adipocytes area **(B)** but also for the adipocytes perimeter **(C)**, equivalent diameter **(D)** and equivalent volume **(E)**; ACL-IFPs samples *n* = 24 and cadaver donors-IFPs *n* = 23 samples. Data are reported as the median and interquartile range (IQR).

The morphometric study was performed for adipocytes characterization and comparison in the two groups (Figure 2; Table 2).

**Table 2:** Infrapatellar fat pad adipocytes morphometric analysis.

**IFP^a^ adipocytes**	**ACL^b^ patients**	**Cadavers**	***p*-value**
	**(*n* = 24)**	**(*n* = 23)**	
Area (μm^2^), mean (range)	1,879.20 (895.94–3,699.15)	3,271.49 (1,801.09–5,339.36)	<0.0001
Major axis (μm), mean (range)	54.47 (39.22–78.56)	73.82 (54.90–100.31)	<0.0001
Minor axis (μm), mean (range)	41.21 (27.20–60.27)	54.66 (39.92–66.98)	<0.0001
Eccentricity, mean (range)	0.65 (0.50–0.75)	0.66 (0.45–0.78)	0.835
Perimeter (μm), mean (range)	158.7 (114.5–234.1)	220.2 (151.3–288.1)	<0.0001
Diameter (μm), mean (range)	46.41 (32.77–67.90)	63.54 (41.77–80.14)	<0.0001
Volume (μm^3^), mean (range)	60,011 (18,480–164,353)	144,981 (38,267–270,228)	<0.0001

a *IFP, infrapatellar fat pad;*

b *ACL, anterior cruciate ligament*.

The ACL-IFPs exhibited a smaller adipocytes area compared to cadaver donors-IFPs with a mean value of 1,879.20 and 3,271.49 μm^2^, respectively (*p* < 0.0001). Consequently, significantly smaller values were also recorded for the adipocytes major and minor axis. Specifically, mean major axis values were 54.47 vs. 73.82 μm (*p* < 0.0001), while mean minor axis values were 41.21 vs. 54.66 μm (*p* < 0.0001) for cadaver donor-IFPs and ACL-IFPs, respectively. No significant differences were observed regarding the eccentricity between the two groups (*p* = 0.835). The perimeter of adipocytes was smaller for ACL-IFPs compared to cadaver donors-IFPs (mean 158.7 vs. 220.2 μm) (*p* < 0.0001) as well as the equivalent diameter (mean 46.41 vs. 63.54 μm) (*p* < 0.0001) and the equivalent volume (mean 60,011 vs. 144,981 μm^3^) (*p* < 0.0001) (Table 2).

### Extracellular Matrix Proteins Characterization Study: Decrease in Collagens and Elastic Fibers Content Along With Age Despite Samples Architecture Maintenance

**Figure 3:**
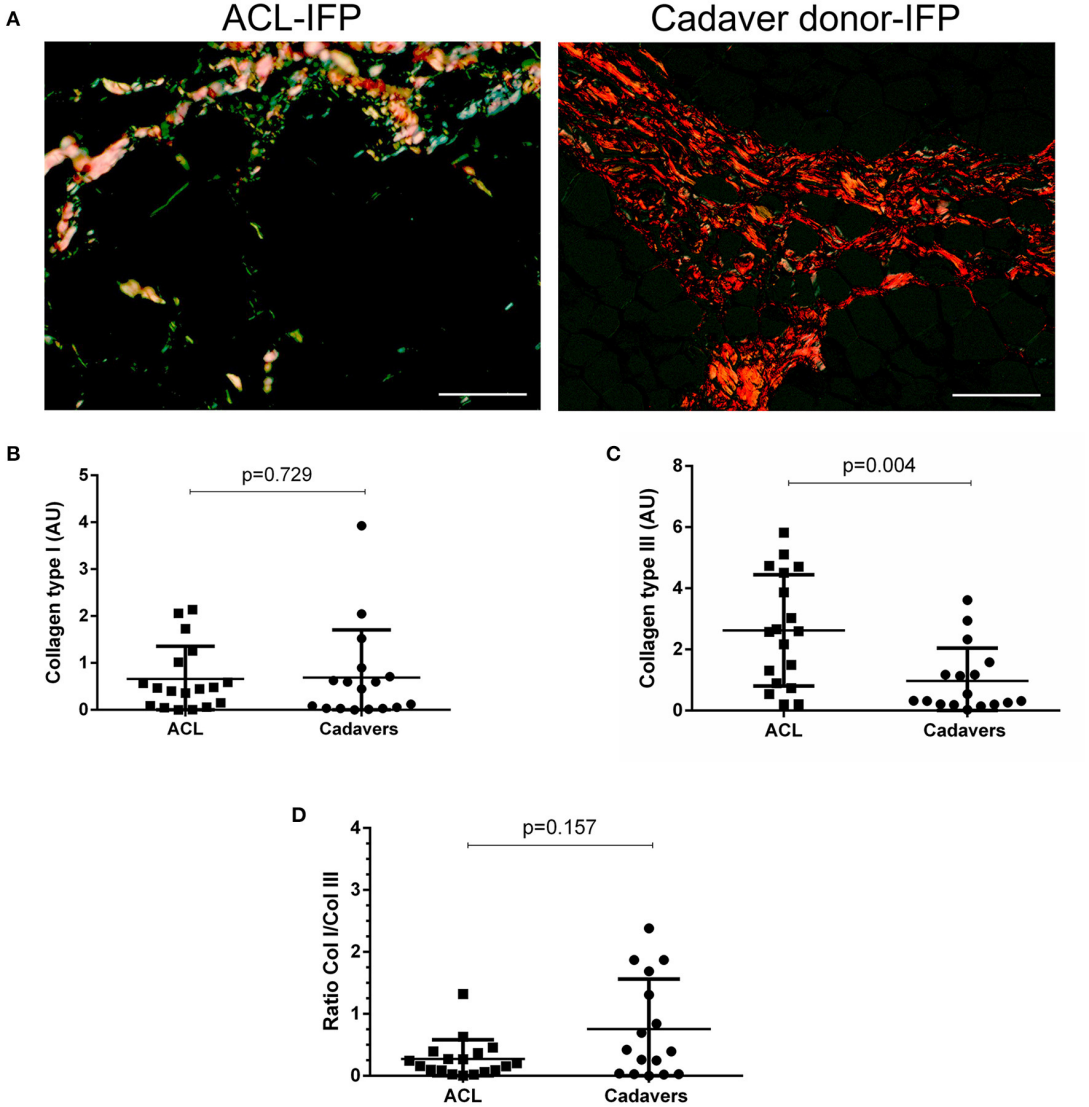
Collagen type I and III distribution. Sirius red stain under polarized light was performed to assess collagen type I (appearing red-orange) and type III (appearing green-yellow) distribution in ACL-IFPs (*n* = 18) **(A)** and cadaver donors-IFPs (*n* = 17) (scale bar = 50 μm). Collagens amount was then calculated in the two groups; any difference was observed in collagen type I content **(B)**; conversely, collagen type III showed significantly higher values for the ACL-IFPs than for cadaver donors (*p* = 0.004) **(C)**. The ratio between the collagens type I/III (Col I/Col III) was then highlighted; any significant difference was observed between the two groups **(D)**. The data analysis considered n=18 tissue samples for ACL-IFPs and *n* = 16 tissue samples for cadaver donor derived-IFPs. Data are reported as the median and IQR. AU, arbitrary unit.

The ECM characterization was focused on collagens (type I and III) and elastic fibers content. Considering collagens, the amount of subtype I was quantitatively similar in the two IFP groups with a median of 0.46 AU (IQR, 1.08–0.08) for ACL-IFPs and 0.45 AU (IQR, 0.80–0.03) for cadaver donors-IFPs (*p* = 0.729); conversely, significant differences were observed for collagen type III, which was higher in ACL-IFPs [median of 2.59 AU (IQR, 4.56–0.86)] than in cadaver donor-IFPs [median of 0.32 AU (IQR, 1.38–0.20)] (*p* = 0.004) (Figure 3). Despite that, the ratio collagen type I/collagen type III was not affected; no difference was detected between the two groups (*p* = 0.157) (Figure 3).

**Figure 4:**
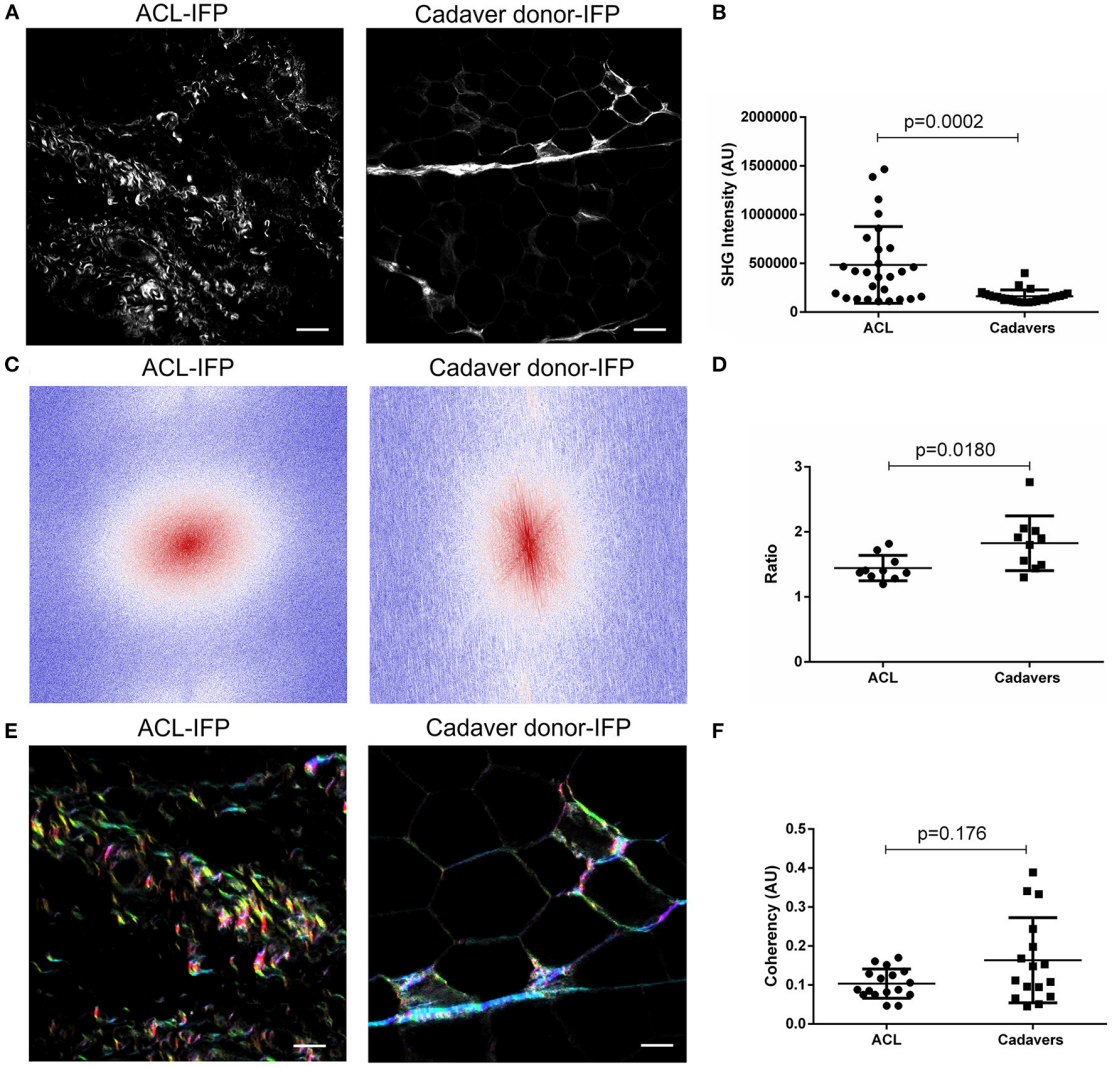
Evaluation of collagen content and distribution. **(A)** Representative images of ACL-IFP and cadaver donor-IFP samples after label-free Second Harmonic Generation (SHG) collagen analysis (scale bar = 50 μm). **(B)** SHG intensity evaluation: a significant difference (*p* = 0.0002) was detected for total collagen content in the cohort, with higher values in the ACL-IFP group than in the cadaver donor-IFP group. **(C)** Fast Fourier Transforms (FFTs) applied to ACL-IFP and cadaver donor-IFP samples showing a circular and an oval collagens orientation for ACL-IFPs and cadaver donor-IFPs, respectively. **(D)** FFT power plots in **(C)** were fitted with an ellipse and the ratio of long and short axis is reported. **(E)** Hue, Saturation and Brightness (HSB) maps obtained through the OrientationJ plugin for the ACL-IFP and cadaver donor-IFP samples; in these maps, to an orientation angle of the collagen is assigned a color and a specific color saturation (scale bar = 100 μm). **(F)** Coherency analysis did not show significant differences in the cohort.

Total collagen content was also investigated by SHG (Figure 4A): SHG mean intensity was higher in ACL samples [median of 408,857 AU (IQR, 111,582–1,465,406)] compared to cadaver-donor samples [median of 147,882 AU (IQR, 100,365–400,824)] (*p* = 0.0002) (Figure 4B).

To deepen the knowledge on collagen architecture in the two sample populations, Fast Fourier Transform (FFT) and OrientationJ were adopted and coherency was determined. For FFT, two representative images are proposed (Figure 4C) showing a different collagen orientation for the groups (i.e., circular and oval for ACL-IFPs and cadaver donor-IFPs, respectively). Thus, FFT power plots in Figure 4C were fitted with an ellipse and the ratio of major and minor axes was determined. The calculated median values were: 1.389 (IQR, 1.587–1.309) and 1.853 (IQR, 2.025–1.481) for ACL samples and cadaver samples, respectively (*p* = 0.0180) (Figure 4D). Furthermore, OrientationJ allowed to visualize the fibers' direction within the sample through HSB map. All colors were represented in the two experimental groups suggesting that collagen did not display a preferential orientation (Figure 4E). The coherency was slightly reduced in ACL-IFP samples; however, any statistical significance was observed between the two groups (Figure 4F).

**Figure 5:**
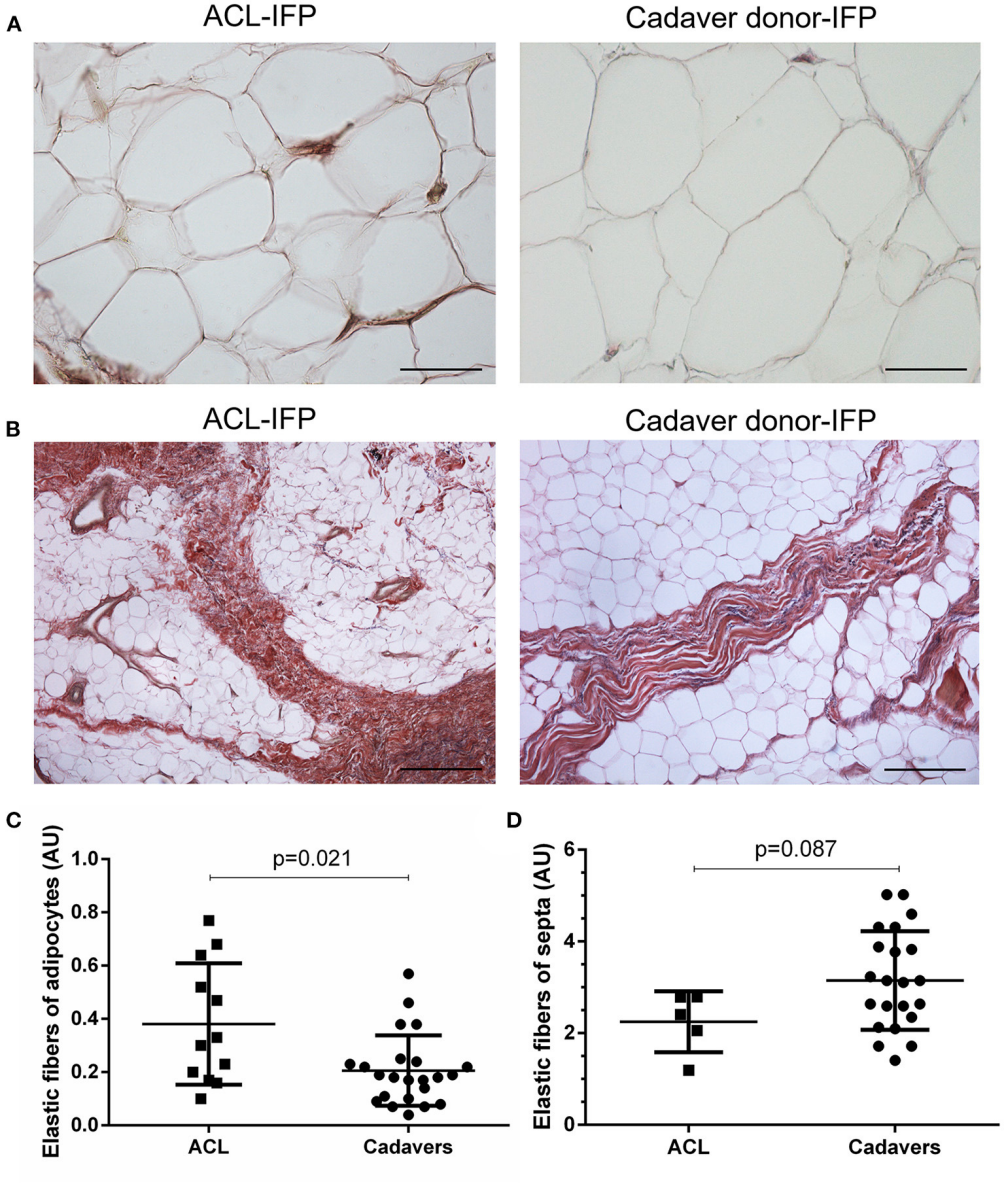
Elastic fibers distribution. Van Gieson staining showed in violet the elastic fibers orientation around the adipocytes **(A)** (scale bar = 200 μm) and in correspondence of the septa **(B)** (scale bar = 50 μm) for ACL-IFPs and cadaver donors-IFPs. Quantification of the elastic fibers showed differences in the two settings. Significantly, higher elastic fibers were observed around the adipocytes in the ACL-IFPs (*p* = 0.021) **(C)** but not in correspondence of the septa in the cadaver donors-IFPs (*p* = 0.0872) **(D)**. For the adipocytes surrounding areas, data analysis considered *n* = 12 and *n* = 23 tissue samples for ACL-IFPs and cadaver donors-IFPs, respectively; for the septa, data analysis considered *n* = 5 and *n* = 23 tissue samples for ACL-IFPs and cadaver donors-IFPs, respectively. AU, arbitrary unit.

Regarding the presence of the elastic fibers, a higher content was recorded in the areas surrounding the adipocytes of ACL-IFPs [median 0.31 AU (0.61–0.18)] and cadaver donor-IFPs [median 0.18 AU (IQR, 0.24–0.10)] (*p* = 0.021) (Figure 5). Moreover, differences were also observed in the elastic fibers of the septa showing lower levels in the ACL-IFP group [median of 2.41 AU (IQR, 2.79–1.62)] than in the cadaver donors-IFP group [median of 3.13 AU (IQR, 4.00–2.29)] (*p* = 0.087) (Figure 5). Due to the small dimension of the ACL-IFP specimens, only 5 samples allowed for the detection of septa.

### Correlations With Age

**Figure 6:**
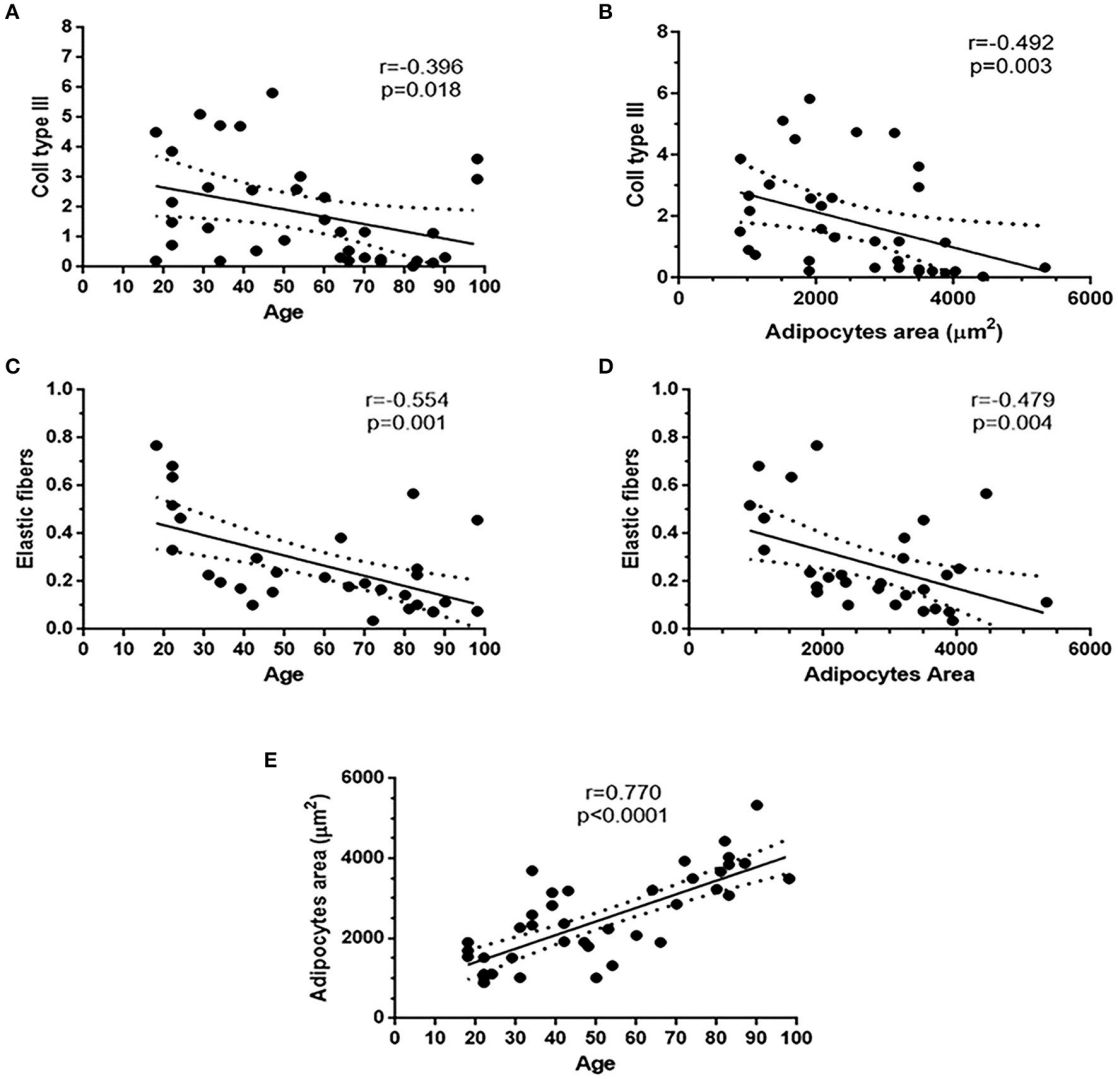
Existing correlations in the overall patient's cohort. Collagen III and elastic fibers content were negatively correlated with age **(A,C)** and adipocytes area **(B,D)**. Adipocytes area was positively correlated with age **(E)**.

Collagen type III of the overall cohort was negatively correlated with age (*r* = −0.396; *p* = 0.018) and with adipocyte area (*r* = −0.492; *p* = 0.003). Elastic fiber content was negatively correlated with age (*r* = −0.554; *p* = 0.001) and with adipocyte area (*r* = −0.479; *p* = 0.004). Age of the overall cohort was positively correlated with the adipocyte area (*r* = 0.770; *p* < 0.0001) (Figures 6A–E). No correlations were found between adipocyte area and BMI.

## Discussion

For the first time to our knowledge, this study allowed to describe the specific characteristics of the IFP tissue with respect to age, in case of no OA signs. Preliminarily, (a) the absence of inflammatory features; (b) an aging-related variation in adipocytes area, despite typical cell morphology maintenance; (c) a reduction in ECM proteins with a possible different distribution with aging, were here hypothesized.

**Figure 7:**
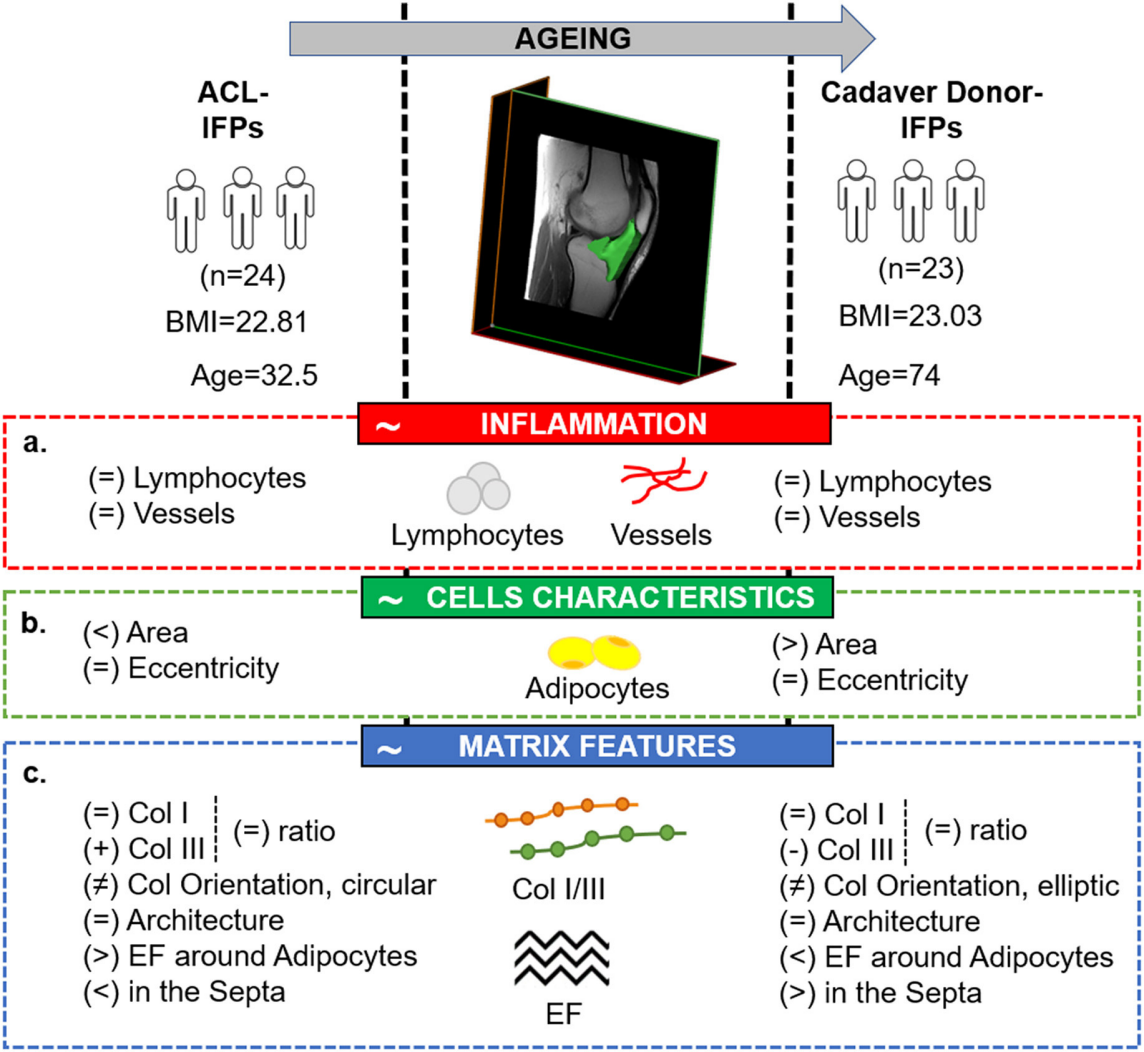
Comparative picture between the two study groups, showing the tissutal specific features related to IFP physiological maturation, in case of the absence of carilage defect. The image highlights the main study findings with respect to the initial hypotheses which have been identified in the red (a. absence of inflammatory features in case of no cartilage lesions), green (b. variation in adipocytes area despite manteinence of the typical cells morphology with aging) and blue (c. reduction in extracellular matrix proteins and different distribution with aging) boxes, respectively. ACL, anterior cruciate ligament reconstruction; BMI; body mass index; Col, collagen; EF, elastic fibers; IFP, infrapatellar fat pad.

After confirmation of no inflammatory signs in all samples, the study results also proved the initial assumptions related to IFP cells and ECM. Experimental data showed that young subjects (ACL donors-IFP group) had smaller adipocytes, a higher content of collagen III, total collagen and elastic fibers around adipocytes compared to elderly patients (cadaver donors-IFP group). On the contrary, the levels of collagen I, ratio of collagens and the architecture of collagen were comparable between the two groups. Negative correlations were found between collagen III, elastic fiber content and age, while a positive correlation was highlighted between adipocytes area and age. Collagen III and elastic fibers negatively correlated with adipocytes area. Thus, aging seems to affect the anato-morphometric characteristics of healthy IFP (Figure 7).

The need for consciousness on physiological IFP changes aging-related, arise from the interest toward OA developmental mechanisms and its clinical manifestations which has expanded in recent years. OA is a multifactorial irreversible disease, even defined as a group of different overlapping distinct joint disorders sharing similar biological, morphological and clinical outcomes, and supported by both systemic and local inflammation leading to pain and loss of joint function (2, 30, 31). Thus, there is consensus in claiming that the correct identification and understanding of the aspects involved in this condition can help to improve the management of OA patients. Awareness on the role of the IFP in OA pathogenesis is recent and intriguing; besides IFP pro-inflammatory phenotype in patients with advanced joint disease (32), many authors also support its dual function in OA onset and progression. In addition to the secretion of inflammatory mediators, it also responds to the pathologic OA-joint environment boosting co-chained/continuous events and going through changes itself (8, 33, 34).

As postulated by Lakatta (18, 35, 36), aging is a shift condition characterized by transformed structures and functions of the body. Through proteome-wide measurements approaches with two-dimensional multiplexing technology, Yu et al. (37) showed that white adipose tissue is the tissue most affected by aging, thus suggesting its importance for the body's adaptation and response to getting older. Hence, to better unravel the role of the IFP in OA, here we focused on IFP physiological remodeling in order to guarantee for adequate key descriptive elements which, in turn, may be useful to distinguish physiological- from disease-related morphological changes in this tissue.

IFP tissues from non-OA young and elderly subjects were compared and non-pathogenicity ascribable to eventual inflammatory process was confirmed by histological evidence. Often, chronic inflammation results in fibrosis which in turn is associated to disruption of the ECM deposition/degradation balance (38); thus, proving tissue quality (i.e., healthy) was a fundamental study prerequisite. In particular, according to the IFP-histopathological grading, any of the OA-signs previously described (10) were observed, in particular, no increased lymphocytic infiltration and/or vascularity in both experimental groups was detected. It is well-known that acute ACL rupture determines an increase of inflammation followed by a decrease of inflammatory mediators' release in the chronic phase (39). Here, we enrolled patients with chronic (more than 6 months) ACL rupture to avoid the inflammation of the acute phase.

Considering our morphometric study data, IFP adipocytes from the two groups displayed an almost superimposable eccentricity, implying the maintenance of the typical cell morphology, while higher area (and greater major and minor axes dimensions), perimeter, equivalent diameter and equivalent volume were detected for the adipocytes of the cadaver donors-IFP group. As the mean BMI was that of normal weight individuals, no morphological correlation was observed with obesity or other related chronic conditions. Rather, aging is likely exerts a role; in fact, in accordance with our correlation analysis (age/adipocytes morphometric data), recent experimental evidence by Gustafson et al. (40) showed that adipose cell size increased significantly with age, a finding that also reflects the *in vitro* data by Zoico et al. (41) on morphological characteristics of 3T3-L1 adipocytes which, along with aging, varied their morphology, acquiring a greater diameter and volume in culture. A positive correlation between adipocytes area and age was also found in our previous study comparing ACL-IFPs with OA-IFPs but age did not have any significant effect on the response variables (inflammation, vascularity, adipocyte numbers and adipocytes area) by generalized linear regression models, demonstrating that these IFP features are affected by OA independently from the impact of age (42). We would need to consider that in our previous study, we have compared not only young vs. older but also OA vs. non-OA (10). In the present study, an age-dependent increase of adipocyte area and the potential links with senescence were observed, thus suggesting that this feature can be part of a physiological process but can also be related to the OA pathology.

A cell cannot be fully investigated without considering its environment (43). Cellular senescence likely descends from age-related changes in the ECM (44), hence, its features are another valuable aspect to consider when focusing on tissue age-related development. As stated by Rittié et al. (45), tissue remodeling may be assessed focusing on variations in collagen matrix content; here, collagen I and III were both specifically analyzed through a histological approach. Collagen I is associated to stiff fibrils, while the collagen III fibrils (expressed in most of the collagen I-containing tissues) (46) are more compliant (47). The maintenance of specific and adequate collagen I/III ratio in tissues is fundamental to guarantee their functional integrity (48). Increasing collagen I/III ratio would determine additional rigidity to the tissue structure which is thought to exert adverse effects on local tissue health (43); in contrast, decreasing collagen I/III ratio would confer more elasticity and flexibility. Modifications in collagens content are generally the expression of an existing condition, which may be physiological or pathological (48). Considering the study cohort, a greater mechanical stimulation of the IFP in the young patients, likely related to an increased physical activity than the elderly group, could be the cause of the higher values of collagen III, as an adaptive behavior; in fact, collagen III is expressed ubiquitously in tensile stress bearing tissues (49). The negative correlation between age and collagen III in the cohort furtherly supports this assumption. Interestingly, both collagens (i.e., type I and III) displayed lower values in OA-IFPs compared to ACL-IFPs (42) and cadaver donors-IFPs, suggesting a wider affection of the ECM proteins content in the disease. Despite the differences in collagen III content according to age, this variation did not significantly affect the collagen I/III ratio which was comparable between the two groups, suggesting the absence of pathogenic features in the overall cohort.

Collagen fibers and bundles can also be visualized in tissues without labeling approaches through detection of SHG signal, a gold standard technique in many ECM studies (50). This strategy is also a candidate for collagen quantification and investigation of its topology and organization (51). SHG intensity was higher in ACL compared to cadaver-donor samples suggesting that elderly IFPs have a lower content of collagens. The analysis of collagen intensity signal is truly relevant; however, it does not give any information on the organization and distribution of the fibers within the sample. Thus, coherency image analysis was performed adopting two complementary strategies for this analysis: FFT and OrientationJ (52). While significantly different collagen fibers orientation was observed, as showed by FFT power plots analysis, no statistical significance was observed for coherency in the cohort; this result suggests that despite the decrease in collagen deposition and enlargement of adipocytes along with age, the global architecture of the samples was not affected.

For the elastic fibers content, lower values were detected in cadaver donors-IFPs than in the ACL-IFPs in peri-adipocytic areas and in the septa, along with age. These data are in accordance with previous findings by Macchi et al. (8) where a reduction in elastic fibers was ascribed to old donors age. A focus on the dynamic role of the IFP in knee kinematics is appropriate to understand the significance of these data; IFP deforms considerably in knee flexo-extension, as previously assessed by ultrasound analysis (22) and by data on finite element analysis (16, 53, 54). Thus, the higher elastic fibers content in ACL-patients may likely represent the resulting expression of higher dumping requirements due to more repetitive and intensive solicitation in young-age; a sort of non-pathogenic adaptive mechanism to loading, like for collagen III.

Age affects/modulates the characteristics of the IFP tissue determining a variation in ECM proteins' content and specific cells' morphometry. In particular, the 3D microenvironment that cells experience *in vivo* is responsible for different cells' behaviors; ECM in mature tissues is not only critical for tissue structure/function but is also responsible for cells behavior including growth, survival, motility, and spatial organization (55, 56). Moreover, also mechanical stimulation (i.e., physical activity/mobility) likely exerts a role in tissue remodeling and contributes to a progressive variation of cell shape and function referring to ECM proteins secretion. According to the study results, cells modifications interestingly occur also in absence of a pathological condition, confirming the ECM leading role in choreographing cell behavior (57). The existence of a cell/ECM cross-talk, responsible for the reciprocal attitude modulation, is a probable event which, in accordance with Ingber (58), may mirror a modification of tissue biological function.

The study main limitation is related to difficulties in the enrollment of a sufficient number of middle-aged patients/donors for reliable statistical analysis and comparisons; in fact, this further samples group would have allowed to obtain intermediate data to follow tissue characteristics along with aging, in a progressive manner. Despite that, our data showed the age impact on the IFP features in case of no pathological condition likely providing a helpful support to studies that focus on IFP active contribution to joint disease onset and progression, like OA. Further investigation is necessary to better unravel the characteristics of the IFP's ECM, also considering that fibrosis has been suggested as possible therapeutic target in OA. Likewise, a specific focus on gender differences may enlighten the differences in OA prevalence.

## Data Availability Statement

The raw data supporting the conclusions of this article will be made available by the authors, without undue reservation.

## Ethics Statement

The studies involving human participants were reviewed and approved by Body Donation Program of the Section of Human Anatomy, University of Padova, according to European, Italian and regional guidelines. Excision was furtherly authorized by the Italian law No. 10 of February 10, 2020, entitled Rules regarding the disposition of one's body and post-mortem tissues for study, training, and scientific research purposes. The patients/participants provided their written informed consent to participate in this study Ethic committee IOR, Prot. Gen. 0042078 Bologna, December 14, 2015. The patients/participants provided their written informed consent to participate in this study.

## Author Contributions

VM, RD, MF, ES, and EB: conceptualization. ES, MC, EB, DG, CF, and VM: methodology. GB and FR: second harmonic generation studies and images analysis. MC, RB-B, EP, DG, CF, EO, and GF: formal analysis. ES, EB, VM, AP, DG, RR, MF, and PR: data curation. ES and EB: writing. VM, RD, and MF: supervision. All authors contributed to the article and approved the submitted version.

## Conflict of Interest

The authors declare that the research was conducted in the absence of any commercial or financial relationships that could be construed as a potential conflict of interest.
